# Fabrication of Silicon Microfluidic Chips for Acoustic Particle Focusing Using Direct Laser Writing

**DOI:** 10.3390/mi11020113

**Published:** 2020-01-21

**Authors:** Anna Fornell, Per Söderbäck, Zhenhua Liu, Milena De Albuquerque Moreira, Maria Tenje

**Affiliations:** Department of Material Science and Engineering, Science for Life Laboratory, Uppsala University, 75121 Uppsala, Sweden; anna.fornell@angstrom.uu.se (A.F.); per.soderback@gmail.com (P.S.); zhenhua.liu@angstrom.uu.se (Z.L.); milenadeam@gmail.com (M.D.A.M.)

**Keywords:** acoustophoresis, acoustofluidics, laser micromachining, microfabrication, particle manipulation, ultrasound

## Abstract

We have developed a fast and simple method for fabricating microfluidic channels in silicon using direct laser writing. The laser microfabrication process was optimised to generate microfluidic channels with vertical walls suitable for acoustic particle focusing by bulk acoustic waves. The width of the acoustic resonance channel was designed to be 380 µm, branching into a trifurcation with 127 µm wide side outlet channels. The optimised settings used to make the microfluidic channels were 50% laser radiation power, 10 kHz pulse frequency and 35 passes. With these settings, six chips could be ablated in 5 h. The microfluidic channels were sealed with a glass wafer using adhesive bonding, diced into individual chips, and a piezoelectric transducer was glued to each chip. With acoustic actuation at 2.03 MHz a half wavelength resonance mode was generated in the microfluidic channel, and polystyrene microparticles (10 µm diameter) were focused along the centre-line of the channel. The presented fabrication process is especially interesting for research purposes as it opens up for rapid prototyping of silicon-glass microfluidic chips for acoustofluidic applications.

## 1. Introduction

Enrichment of cells and other microparticles is a standard process in bioanalytic laboratories. Similarly, there is a need in microfluidic systems to integrate such operations in order to perform complex biological analysis on-chip. Many different technical solutions to sort, separate and enrich particles in microfluidic systems have been presented including acoustophoresis, deterministic lateral displacement, dielectrophoresis, filters, inertial focusing and magnetophoresis [1,2]. In this work, we have opted for using bulk acoustic waves for particle enrichment.

Acoustic particle focusing has many advantages including high particle enrichment [3], no need for labelling of the sample, biocompatibility [4] and on-demand operation. Acoustofluidic devices are grouped into two categories depending on how the acoustic waves are propagating in the device: surface acoustic wave devices and bulk acoustic wave devices. Typically, bulk acoustic wave devices can be operated at higher flow rates and have simpler fabrication processes, as these devices do not require integration of electrodes serving as interdigital transducers. In bulk acoustic wave devices, the acoustic waves are instead generated by an external piezoelectric transducer that is glued or clamped to the microfluidic chip and the channel acts as an acoustic resonance chamber [5]. Therefore, the channel should be fabricated in a material with high acoustic impedance to allow for the build-up of a strong acoustic standing wave field in the channel. Generally, bulk acoustic wave devices are fabricated in silicon [6,7] or glass [8], both having much higher acoustic impedance (19.8 MRayl and 12.6 MRayl, respectively) than water (1.5 MRayl) [5]. Microfabrication of silicon and glass is commonly done by wet- or dry-etching. Both these processes require many process steps such as mask fabrication, UV-photolithography to define the structures, wet-etching using hazardous chemicals or dry-etching using advanced equipment, and finally bonding the structures using anodic or fusion bonding. In addition, all of these steps have to be performed in a clean room to prevent dust particles interfering with the processes. Therefore, there is a drive in finding new fabrication methods and materials to decrease the cost and time for fabrication of bulk acoustic wave devices. There are a few reports of bulk acoustic wave devices fabricated in plastics [9,10]. Plastic microfluidic chips have the advantage of being suitable for mass production. However, the acoustic impedance difference between plastics such as polystyrene (2.5 MRayl) and water (1.5 MRayl) is much smaller than between silicon (19.8 MRayl) or glass (12.4 MRayl) and water (1.5 MRayl), thus higher actuation voltage of the transducer is required to generate a strong standing wave field in the channel. The higher actuation voltage results in a higher temperature rise in the chip and therefore efficient cooling systems need to be integrated, which complicates the experimental setup. There are also a few reports on bulk acoustic wave devices fabricated in metals such as aluminium and stainless steel [11,12]. However, as of today silicon and glass are still the most commonly used materials for bulk acoustic wave devices.

The aim of this work is to develop a faster and simpler process for fabricating silicon microfluidic chips suitable for particle focusing by bulk acoustic waves. Briefly, in our method the channels were first fabricated on a silicon wafer using a direct laser writing system and the channels were then sealed by a glass wafer using adhesive bonding.

Compared with conventional silicon processing methods laser micromachining omits several process steps, which saves time and simplifies the fabrication process. For example, in laser micromachining the structures are directly written on the silicon wafer thus no mask is needed to define the structures by UV-photolithography. Fabrication of a mask typically takes one day if done in-house or longer if the mask is ordered from an external company. Wet-etching of silicon is typically done with KOH which includes several process steps such as SiO_2_ growth (1 day), UV-photolithography, SiO_2_ etching with HF and Si etching with KOH (a couple of hours) and manually drilling inlet and outlet holes. Dry-etching of silicon is faster than wet-etching and requires less process steps, however still the structures need to be defined using UV-photolithography. Another advantage of laser micromachining is that structures with vertical side walls can more easily be fabricated. Wet-etching of silicon with KOH is highly dependent on the crystal plane orientation of the wafer. Dry-etching of silicon avoids the crystal plane dependence but instead results in uneven side wall profiles due to the repeated cycles of etching and passivation steps in the BOSCH process [13]. Moreover, less equipment and hazardous chemicals are needed in laser micromachining. A comparison between the methods is presented in Table 1.

Laser micromachining is a well-established technology within manufacturing industry for production of microdevices [14], but it is also a suitable technology for rapid prototyping of microfluidic chips in research [15,16]. In addition to fabricating channels, laser processing can also be used to change the wetting properties of the channels [17].

Laser ablation can be done using many types of laser sources with different wavelengths and pulse lengths (nanosecond-, picosecond- and femtosecond lasers) [14]. In this work a Nd:YVO_4_ nanosecond laser source with a wavelength of 532 nm was used. We selected this laser source as it is cheaper than picosecond- and femtosecond laser systems. The principle of laser micromachining is that an intense laser beam interacts with the material and causes ablation if the intensity is above the threshold value for that particular material. The mechanism causing removal of material depends on the interaction time between the material and the laser beam (i.e., if a nanosecond-, picosecond- or femtosecond laser is used). For nanosecond lasers, it is mainly a photothermal process that causes ablation of the material [14]. 

Much work on laser ablation of microfluidic structures has been focusing on polymer systems [15,16]. Klank et al. used a commercial CO_2_ laser system for micromachining of microfluidic channels in polymethylmetacrylate (PMMA) [18]. Laser micromachining of polymers can also be done using other laser systems, for example Suriano et al. investigated laser ablation in polymers using a femtosecond laser system [19]. However, laser micromachining is not limited to polymers but can also be used to make microfluidic structures in other materials [20,21]. Sugioka et al. showed three-dimensional microfluidic structures fabricated in glass using a femtosecond laser system [22]. There are also several reports of laser micromachining of silicon [21,23,24,25,26]. For example Kam et al. fabricated microchannels on a silicon wafer using a femtosecond laser and used the silicon wafer as a mould for polymethylsiloxane (PDMS) moulding [21]. However, silicon microfluidic chips for acoustofluidic applications have not been fabricated using laser micromachining before.

The first part of this work was to optimise the laser process and fabricate the microfluidic chips. The microfluidic chips were then tested for acoustic particle focusing. We show that acoustofluidic devices can be fabricated in 1 day with our method.

## 2. Materials and Methods 

### 2.1. Microfluidic Chip Design

**Figure 1:**
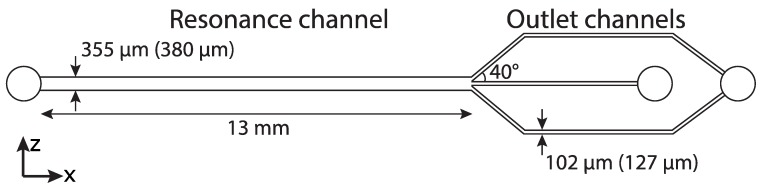
The design of the microfluidic chip. The first values are the values in the CAD drawing, where the values in brackets are the targeted values.

The purpose of this project was to fabricate an acoustofluidic chip suitable for focusing and enrichment of bioparticles such as plastic microbeads or cells. The design of the microfluidic chip consisted of a straight main channel that branched into a trifurcation, Figure 1. The particle solution was introduced in the main channel and the particles were focused by the acoustics along the centre-line of the channel, and the particles were then extracted into the centre outlet channel. 

To allow for acoustic focusing of the particles, half wavelength acoustic resonance was set between the channel walls. Particles in a half wavelength acoustic standing wave field are focused by the primary acoustic radiation force to the pressure nodal line (here the centre-line of the channel). The primary acoustic radiation force on a particle in a 1D planar standing *λ*/2 acoustic field is given by:

(1) $$F^{\mathrm{rad}}=4\pi \Phi \left(\tilde{\kappa },\tilde{\rho }\right)ka^{3}E_{\mathrm{ac}}\sin\left(2kz\right)$$

(2) $$\Phi \left(\tilde{\kappa },\tilde{\rho }\right)=\frac{1}{3}\left[\frac{5\tilde{\rho }-2}{2\tilde{\rho }+1}-\tilde{\kappa }\right]$$

where *λ* is the wavelength of the sound, Φ is the acoustic contrast factor, $\tilde{\kappa }$ is the compressibility ratio between the particle and the fluid, $\tilde{\rho }$ is the density ratio between the particle and the fluid, k is the wavenumber (k=2π/λ), a is the particle radius, $E_{\mathrm{ac}}$ is the acoustic energy density and z is the distance from the channel wall [27].

The targeted final width of the resonance channel was 380 µm to allow for operation with a 2 MHz piezoelectric transducer at the fundamental resonance frequency according to:

(3) $$w=\frac{c}{2f}$$

where *w* is the width of the channel, *c* is the speed of sound of the fluid and *f* is the frequency. The targeted final width of the outlet channels was 127 µm. To compensate for the broadening of the structures in the ablation process the width of all the channels was decreased by 25 µm in the CAD drawing (Section 3.1). The targeted final depth of all the channels was 150 µm, except for the inlet and outlet holes that were ablated through the silicon wafer.

### 2.2. Microfabrication and Process Characterisation

A commercial laser marking system AIO (Östling, Solingen, Germany) equipped with a Nd:YVO_4_ nanosecond laser (532 nm) was used to make the structures. The maximum pulse energy of the laser is 400 µJ and the maximum peak power is 200 kW. The laser beam intensity has a Gaussian distribution, and a lens giving a minimum spot size of 16 µm was used. The system has a stationery height-adjustable worktable, and mirrors are used to deflect and guide the laser beam over the wafer. 

The structures were designed in SolidWorks and the file was imported to the laser marker software (XS Designer, Solingen, Germany). The structures were fabricated on a single side polished 525 µm thick silicon wafer (Microchemicals, Ulm, Germany). The silicon wafer was placed on the worktable and the focus of the laser was manually adjusted.

In the initial tests the laser process was optimised to find suitable settings for fabrication of a rectangular microfluidic channel with a cross-section of 380 × 150 µm^2^ (w × h) with vertical walls and a flat bottom surface. In the first set of tests the effect of the power of the laser radiation and the pulse frequency was investigated. Four different power levels (50%, 60%, 70% and 80%) and three different frequencies (10 kHz, 50 kHz and 90 kHz) were evaluated. The other laser settings were: 200 mm/s scan speed, bidirectional fill, single lines, fill space of 0.01 mm and 50 passes. 

A test to measure the ablation rate at the optimised laser settings found in the initial tests (50% power and 10 kHz pulse frequency) was done. The other laser settings were: 60 mm/s scan speed, bidirectional fill, triple lines and fill space of 0.01 mm. The number of passes was varied between 25 and 35 with a step size of 1. Evaluation of the broadening of structures after 35 passes were done by designing and fabricating structures with a width between 355–380 µm and 92–127 µm.

A test to study the effect of the position of the structures relatively to the laser was performed. The structures were positioned −40°, 0° and +40° relative to the laser. The laser settings were: 50% power, 10 kHz pulse frequency, 60 mm/s scan speed, bidirectional fill, triple lines, fill space of 0.01 mm and 35 passes.

Images of the structures were acquired using a SEM (1530, Zeiss, Oberkochen, Germany). Cut-section maps of the structures were acquired by optical profilometry (Nexview^TM^NX2, Zygo, Middlefield, CT, USA).

### 2.3. Fabrication of the Microfluidic Chip

The microfluidic chip was fabricated with the design shown in Figure 1 and with the method described in Section 2.2. The settings for making the channels were: 50% power, 10 kHz pulse frequency, 60 mm/s scan speed, bidirectional fill, triple lines, fill space of 0.01 mm and 35 passes. The inlet and outlet holes were made using the same laser settings except that 150 passes were required to ablate through the wafer. 

The silicon wafer was bonded to a 725 µm thick borosilicate glass wafer (Microchemicals) using Ormocomp resist (Micro Resist Technology, Berlin, Germany) as an adhesive layer. The silicon wafer was first treated in buffered HF for 1 h. Next, both the silicon wafer and the glass wafer were cleaned in acetone in an ultrasound bath for 1 min, rinsed with acetone and isopropanol, followed by cleaning in a plasma asher for 1 min. Then, 1 mL of Ormocomp resist was spin coated onto the glass wafer following the supplier’s instructions. The Ormocomp was partially cured by UV light exposure in a mask aligner (40 s) to avoid that uncured resist fills the channels. The silicon wafer and the Ormocomp coated glass wafer were then placed in contact and fully cured by UV light exposure (90 s). The bonding process was completed with a final baking step at 130° C for 30 min.

The bonded silicon-glass wafer was cut into individual chips using a dicing saw (DAD 361, Disco, Tokyo, Japan). On the silicon side of the microfluidic chip a 4 × 8 mm^2^ piezoelectric transducer resonant at 2 MHz (Pz 26 material, Ferroperm Piezoceramic, Kvistgård, Denmark) was attached using a thin layer of cyanoacrylate glue (Loctite 420, Henkel, Düsseldorf, Germany). One centimetre long pieces of silicone tubing were attached on the inlet and outlet holes using silicone adhesive (Elastosil A07, Wacker, Munich, Germany). 

### 2.4. Acoustic Experiments

**Figure 2:**
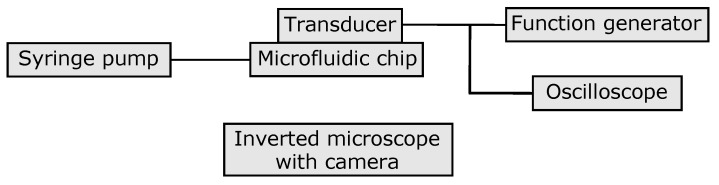
The experimental setup.

An overview of the experimental setup is shown in Figure 2. The microfluidic chip was mounted in a 3D printed holder and placed in an inverted microscope (TE2000-U, Nikon, Tokyo, Japan). The images were acquired using a CMOS camera (DFK NME33UX174, The imaging Source, Bremen, Germany). The microfluidic chip was connected to three 1 mL plastic syringes via polyethylene tubing (0.38 mm inner diameter, BD Intramedic PE Tubing, Becton, Dickinson and Company, Franklin Lakes, NJ, USA). The syringes were mounted on three syringe pump modules (Nemesys, Cetoni, Korbussen, Germany.). Polystyrene particles (10 µm Fluoro-Max green fluorescent particles, ThermoFisher Scientific, Waltham, MA, USA) were suspended in deionized water with 0.01% Triton-X (Sigma-Aldrich, St. Louis, MO, USA). The particle concentration in the original solution was 8 × 10^5^ particles/mL. The inlet flow rate was set to 20 µL/min corresponding to an average flow speed of 5.8 mm/s in the resonance channel. The centre outlet flow rate was set to 5 µL/min and the common side channel outlet flow rate was set to 15 µL/min. 

The piezoelectric transducer was actuated by a function generator (AFG3022C, Tektronix, Beaverton, OR, USA) and the voltage over the piezoelectric transducer was measured by an oscilloscope (TBS1102B, Tektronix). The acoustic actuation frequency was selected to be 2.03 MHz based on strong focusing effect observed in the chip at that frequency. The amplitude of the applied signal was 10 V_peak-peak_. 

The recorded videos were analysed in ImageJ by averaging 400 frames from the video sequence (5 s), calculating the background image as the minimum intensity frame, and then subtracting the background image from the average intensity image.

## 3. Results and Discussion

### 3.1. Characterisation of the Laser Micromachining Process

**Figure 3:**
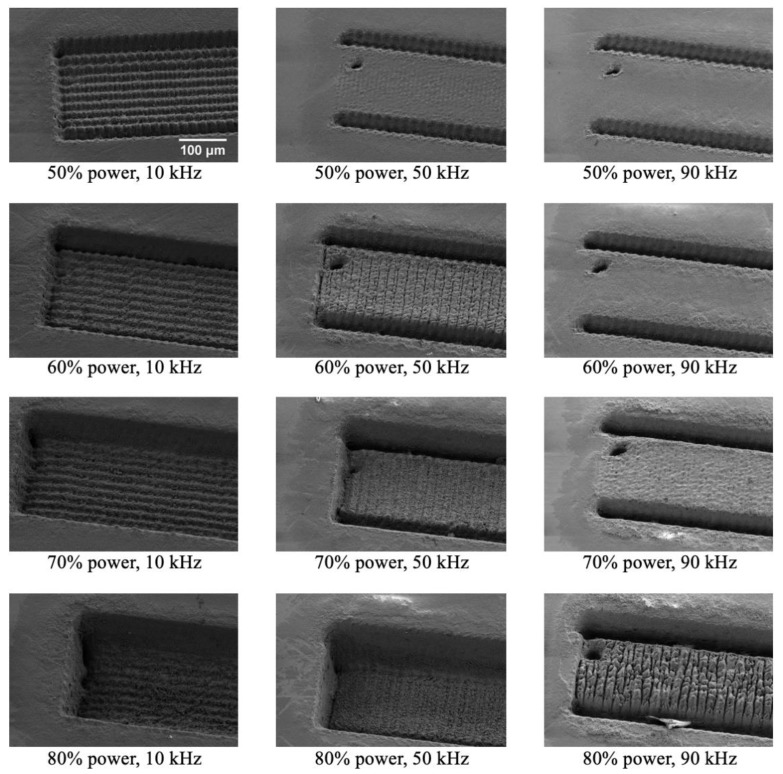
SEM images of structures fabricated at different laser radiation powers and pulse frequencies. In all images the number of passes are 50, and all images are the same scale.

The power and pulse frequency of the laser have a large impact on the quality of the structures, and an initial test to find suitable settings for the fabrication of the microfluidic chip was performed (Figure 3). In this work the aim was to fabricate structures with vertical walls and a flat and smooth bottom surface. 

The intensity of the laser spot has a Gaussian distribution, and for ablation to occur the intensity must exceed a certain threshold value. Generally, a low pulse frequency is good as that gives a shorter pulse duration due to the laser characteristics. For a given energy it is advantageous to have a short pulse duration as this more efficiently uses the delivered energy for ablation instead of heating of the material. Depending on the power settings, material could be ablated at all tested pulse frequencies (10 kHz, 50 kHz and 90 kHz). However, the most uniform structures at different power settings were observed at 10 kHz, and therefore the pulse frequency was set to 10 kHz for the fabrication of the microfluidic chips. Having a low pulse frequency at high power settings might damage the laser, therefore the lowest tested pulse frequency was 10 kHz.

The effect of power on the ablation of the structures was tested. As expected, the depth of the structures was increased with increased power (the number of passes was kept constant). At a pulse frequency of 10 kHz the structures were successfully ablated between 50% and 80% power. A power of 50% was chosen for the fabrication of the microfluidic chip as lower power gives a gentler process. However, it has the disadvantage that the fabrication time is longer.

One important parameter to control is the ablation rate as this determines the depth of the structures. From the initial tests (data not shown) it was determined, at the present laser settings, that 35 passes were required for making structures with a depth of 150 µm. In the initial tests, it was also observed that the ablated structures were broader than what was designed in the CAD drawing. The width of the structures is crucial to control as half wavelength resonance must be obtained between the channel walls to generate the acoustic focusing effect. Based on the initial results (data not shown) it was determined to reduce the width of all the structures by 25 µm in the CAD drawing to compensate for the broadening effect from the machining. 

**Figure 4:**
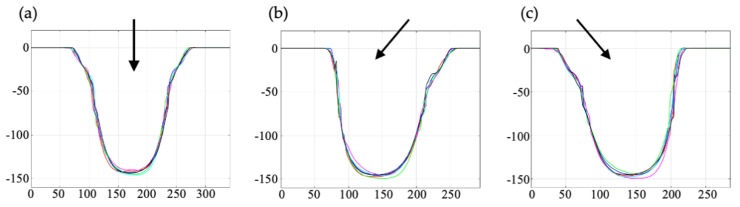
Cut-section profiles of the outlet channels that are placed 0° and ±40° relatively to the laser axis. (**a**) Aligning the structures with the laser axis results in symmetric walls. (**b**,**c**) Structures placed not aligned with the laser axis (±40°) will be asymmetric where the wall facing the laser will have a steeper slope than the wall facing away from the laser axis. The arrow indicates the direction of the laser. Six structures for each position were evaluated, and all values are in µm.

In the laser system used in this work, mirrors are used to deflect the laser beam over the wafer and consequently the angle of attack of the laser varies depending on the positioning of the structures on the worktable. In the tests, it was observed that if the structures were aligned with the laser axis the left walls and the right walls became symmetric (Figure 4a). However, when the structures were not aligned with the laser axis (Figure 4b,c) the left walls and the right walls were not symmetric. For non-aligned structures the walls facing the laser have a steeper slope than the walls facing away from the laser. It was decided that the resonance channel should be aligned with the laser axis as it is important that the resonance channel has symmetric and vertical walls.

### 3.2. Evaluation of the Microfluidic Chips

**Figure 5:**
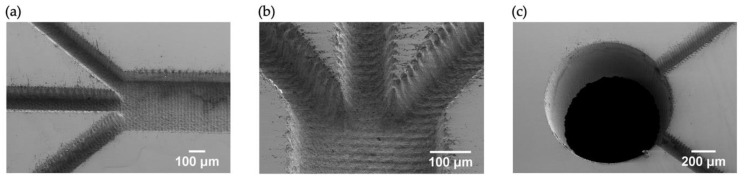
(**a**,**b**) SEM images of the microfluidic channel. (**c**) SEM image of the outlet hole.

Microfluidic channels were fabricated, and SEM images of the channels and one of the outlet holes are shown in Figure 5. At the walls and the bottom of the structures striations could be observed. Additionally, a lip of recast material can be observed at the outer border of the structure. This could be problematic as it makes proper bonding of the structures more difficult.

**Figure 6:**
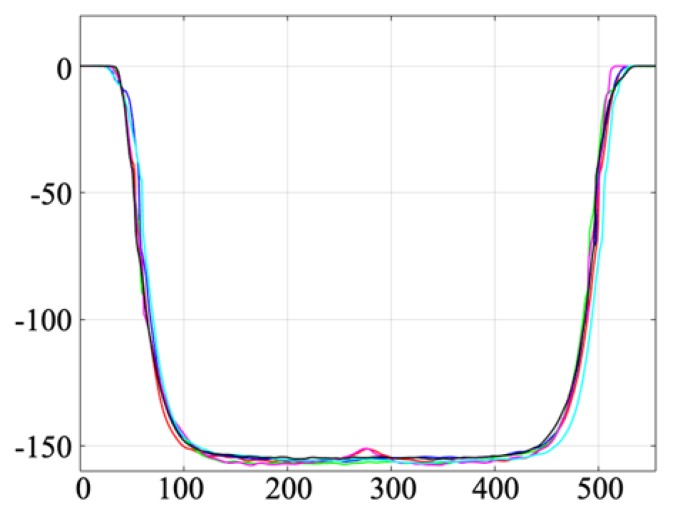
The cut-section profile of the resonance channels. Six structures were evaluated, and all values are in µm.

The cut-section profiles of six resonance channels are shown in Figure 6, and the cut-section profiles for the outlet channels are the same as those shown in Figure 4 (a: centre outlet channel and b,c: side outlet channels). For the outlet channels (Figure 4) only a small part of the bottom surface is flat, thus indicating the lower limit of how small structures that can be fabricated with this depth, using the present laser settings if vertical walls are required. However, it should be noted that in the current design it is only required that the resonance channel has vertical walls. The widths of the resonance channel at 5% and 50% depth were 387.6 ± 2.7 µm (s.d.) and 352.3 ± 2.0 µm (s.d.) respectively, and for the outlet channels the corresponding values were 143.8 ± 7.4 µm (s.d.) and 98.9 ± 2.1 µm (s.d.). The average depth of the resonance channel was 156.6 ± 0.8 µm (s.d) and the average depth of the outlet channels was 145.3 ± 2.2 µm (s.d.).

**Figure 7:**
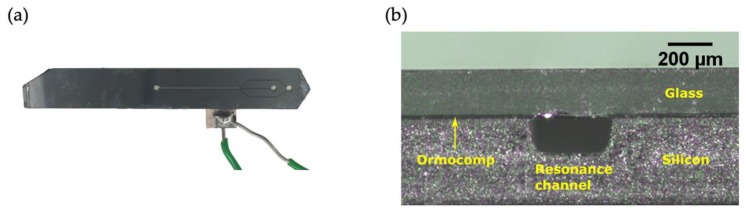
(**a**) The microfluidic chip. (**b**) Cross-section of the resonance channel.

The standard process to bond silicon and glass is by anodic bonding. In this work we propose an alternative method where the structured silicon wafer is bonded to a glass wafer using a thin layer of UV curable hybrid polymer (Ormocomp). In Figure 7, a photograph of a fully assembled chip and the cut-section of the resonance channel is shown. The thickness of the Ormocomp layer was 20 µm. By using Ormocomp bonding there is less strict requirements on the surface roughness and the cleanness of the wafers compared with anodic bonding. This is advantageous because the ablation process often results in recast material and particle contamination over the wafer (Figure 5). In addition, the bonding process is faster than anodic bonding. For comparison, one laser ablated silicon wafer was bonded using standard anodic bonding (data not shown), but that resulted in a significantly lower yield of properly bonded chips.

### 3.3. Acoustic Experiments

**Figure 8:**
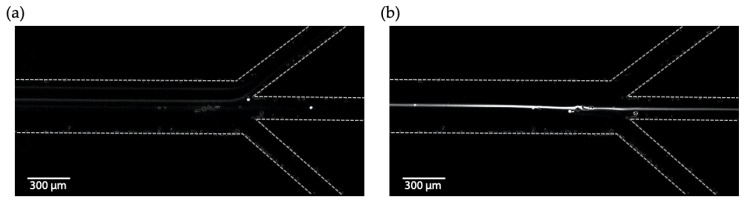
Acoustic focusing of 10 µm fluorescent particles. (**a**) Without acoustics the particles are not focused (hence hard to see in the fluorescent image) and do exist in both the centre and side outlet channels. (**b**) With the acoustics the particles are focused along the centre-line of the resonance channel and enriched in the centre outlet channel. The dashed lines show the borders of the channels.

The microfluidic chip was assembled and an experiment to test acoustic particle focusing in the chip was done. Half wavelength resonance was set in the channel and fluid containing particles was injected. With the acoustics applied, the particles were focused along the centre-line of resonance channel, Figure 8. The best focusing was observed at 2.03 MHz. The results show that the Ormocomp bonding layer did not seriously dampen the acoustics as it is possible to obtain strong particle focusing at a commonly used voltage level in acoustofluidic experiments (10 V_peak-peak_). At the trifurcation, 1/4 of the fluid was withdrawn in the centre outlet channel (5 µL/min) while the rest was withdrawn in the side outlet channels (15 µL/min). The acoustic focusing caused the majority of the particles to be directed and enriched in the centre outlet channel. This experiment shows the possibility to use a laser micromachined silicon-glass microfluidic chip for acoustic particle focusing. As the fabrication method is faster compared with conventional silicon processing methods it will open up for rapid prototyping and testing of new channel designs.

## 4. Conclusions

In this work, we have investigated the use of a nanosecond direct writing laser system for the fabrication of microfluidic channels in silicon suitable for acoustic particle focusing. The power and pulse frequency of the laser were optimised, and the number of passes required to make 150 µm deep channels was determined. After the optimisation process, it was possible to make silicon channels with a cross-section of 380 × 150 µm^2^ (w × h) with vertical walls and a flat bottom surface. The microfluidic channels were sealed with a glass wafer using adhesive bonding with Ormocomp resist. The design of the microfluidic chips consisted of a resonance channel that branched into a trifurcation outlet. With acoustic actuation at 2.03 MHz, a half wavelength acoustic standing wave field was generated in the resonance channel and polystyrene microparticles were focused into the centre outlet channel. We think that the presented fabrication method will be useful for rapid prototyping of acoustofluidic devices.

## Figures and Tables

**Table 1:** Comparison between conventional silicon processing and laser micromachining.

Criteria	Wet-Etching with KOH	Dry-Etching Using BOSCH Method	Laser Micromachining
**Required equipment**	mask writer, oven for growth of SiO_2_, O_2_ plasma oven, spinner, hotplate, mask aligner, wet-bench, drilling tool	mask writer, spinner, hotplate, mask aligner, wet-bench, dry-etcher, drilling tool	laser marking system
**Equipment cost**	+++	+++	++
**Hazardous chemicals**	photoresist, HF, KOH	photoresist	none
**Design possibilities**	restricted by the Si crystal planes	free	free
**Time**	+++	++	+
